# P03-004 - Production of proinflammatory cytokines in PFAPA

**DOI:** 10.1186/1546-0096-11-S1-A199

**Published:** 2013-11-08

**Authors:** A Kozlova, O Barabanova, N Davydova, A Shcherbina

**Affiliations:** 1 Immunology, Federal Research and Clinical Center of Pediatric Hematology, Moscow, Russian Federation; 2 Immunology, Children's Clinical Hospital N9, Moscow, Russian Federation

## Introduction

The PFAPA syndrome, associated with aphthous stomatitis, pharyngitis and cervical adenitis which is characterized by monthly flare-ups of fever is quite common in early childhood. Affected children experience their first attack before the age of 5, with fever episodes usually abating in adolescence or young adulthood. Usually a single administration of oral corticosteroids aborts attacks. Tonsillectomy is successful in the prevention of recurrence of further episodes. To date, the cause of this syndrome is unknown. Several studies demonstrated increased serum levels of proinflammatory cytokines(interleukin 6, interleukin 1b) in these patients during the attacks.

## Objectives

Determining the level of intracellular proinflammatory cytokines in patients with PFAPA syndrome during and between the attacks compared to the level of these cytokines in the control group

## Methods

Four patients with PFAPA syndrome were studied during and outside febrile episodes. We determined intracellular cytokines ( IL-1β, IL6, IL8, TNFa) in resting and LPS-stimulated monocytes

## Results

Not surprisingly, and concurrent with previous publications during the PFAPA attack we saw increased basal levels of IL1, which rose somewhat upon LPS stimulation. Basal level of IL6 was also increased during the attack and did not respond to LPS stimulation. TNF level in PFAPA patients with or without attacks was not significantly different from the control group. Interestingly, intracellular levels of the proinflammatory chemokine IL8 was drastically decreased in PFAPA patients during the attack.

## Conclusion

During the attack of PFAPA there is a significant increase in IL1 production, which is concurrent with systemic symptoms during the flare-ups and makes use of IL1 inhibitor drugs potentially effective in the treatment of the flares. Decreased IL8 levels might represent abnormal regulation of inflammation in PFAPA and require further investigation.

## Competing interests

None Declared.

